# Male courtship preference during seasonal sympatry may maintain population divergence

**DOI:** 10.1002/ece3.4640

**Published:** 2018-11-22

**Authors:** Abigail A. Kimmitt, Samantha L. Dietz, Dustin G. Reichard, Ellen D. Ketterson

**Affiliations:** ^1^ Department of Biology Indiana University Bloomington Indiana; ^2^ Department of Biological Sciences North Carolina State University Raleigh North Carolina; ^3^ Department of Biological Science Florida State University Tallahassee Florida; ^4^ Department of Zoology Ohio Wesleyan University Delaware Ohio

**Keywords:** mate choice, migration, population divergence, seasonal sympatry

## Abstract

Animal migration can lead to a population distribution known as seasonal sympatry, in which closely‐related migrant and resident populations of the same species co‐occur in sympatry during part of the year, but are otherwise allopatric. During seasonal sympatry in early spring, residents may initiate reproduction before migrants depart, presenting an opportunity for gene flow. Differences in reproductive timing between migrant and resident populations may favor residents that exhibit preferences for potential mates of similar migratory behavior and reproductive timing, thus maintaining population divergence. We studied dark‐eyed juncos (*Junco hyemalis*), a songbird that exhibits seasonal sympatry. We conducted simulated courtship interactions in which we presented free‐living resident males with either a caged migrant or resident female and quantified courtship behavior prior to the departure of the migrants. We found that resident males preferred to court resident females: they sang more short‐range songs and exhibited more visual displays associated with courtship when presented with resident females. We conclude that males distinguish between migrant and resident females during seasonal sympatry when the risk of interacting with non‐reproductive, migrant females is high. Male mate choice in seasonal sympatry is likely adaptive for male reproductive success. As a secondary effect, male mating preference could act to maintain or promote divergence between populations that differ in migratory strategy.

## 1 INTRODUCTION

The most widely supported models of speciation propose that populations diverge in allopatry, when they are geographically, and therefore reproductively, isolated. However, differentiation between populations that are not geographically isolated is common (Bolnick & Fitzpatrick, 2007; Kirkpatrick & Ravigné, 2002; Kopp et al., 2018; Papadopulos et al., 2011), especially in migratory animals (Winker, 2010). Differences in migratory behavior can lead to sympatry between diverging populations during part of the year so that populations are not fully allopatric (Rolland, Jiguet, Jønsson, Condamine, & Morlon, 2014; Winker, 2010). Many migratory lineages, particularly in birds, have diversified despite the potential for increased dispersal and gene flow associated with a migratory life history. Thus, the isolating mechanisms underlying the divergence of migratory species remain poorly understood and migratory birds represent an ideal system for exploring how speciation can occur in the absence of complete allopatry.

Comparisons of closely‐related populations (ranging from populations within species to sister species) of migratory birds have found that these populations tend to exhibit low rates of phenotypic divergence (e.g., song, plumage, and size) (Delmore, Kenyon, Germain, & Irwin, 2015; Turbek, Scordato, & Safran, 2017; Winker, 2010), suggesting that neither local adaptation in allopatry nor assortative mating primarily drives divergence. The divergence and speciation may be primarily driven by differences in migratory behavior (Delmore et al., 2015; Friesen, Burg, & McCoy, 2007; Irwin & Irwin, 2005; Rohwer & Irwin, 2011; Webster & Marra, 2005). Specifically, differences in migratory behavior can cause reproductive allochrony, in which populations differ in breeding phenology (Winker, 2010). Even when populations are not geographically isolated, they may be temporally isolated. The allopatric model of speciation does not adequately consider how allochrony explains differentiation between migratory populations, as the model does not consider annual shifts in distributions (Winker, 2010).

Divergence in migratory behavior, and consequently reproductive timing, may lead to a special case of temporal divergence known as heteropatry or seasonal sympatry (Fudickar et al., 2016; Ketterson, Fudickar, Atwell, & Greives, 2015; Winker, 2010; Winker, McCracken, Gibson, & Peters, 2013; Withrow, Sealy, & Winker, 2014). Seasonal sympatry refers to situations in which closely related migratory and sedentary animal populations co‐exist during part of the year but are allopatric at other times of year. Reproductive allochrony may drive divergence in seasonally sympatric populations that are overlapping in distribution in early spring. In obligate migrants, spring migration likely constrains the initiation of reproduction, as physiological changes necessary to prepare for long‐distance flight are energetically demanding (Ketterson et al., 2015; Ramenofsky & Nemeth, 2014; Ramenofsky & Wingfield, 2017; Ramenofsky, Cornelius, & Helm, 2012). On the other hand, sedentary individuals are likely able to rely more heavily on supplementary cues and initiate breeding when conditions are optimal (Caro et al., 2009; Ketterson et al., 2015; Robinson et al., 2009; Visser, Caro, Oers, Schaper, & Helm, 2010).

If migratory and sedentary populations overlap in distribution, reproductive allochrony may directly prevent interbreeding (Friesen et al., 2007; Kopp et al., 2018; Taylor & Friesen, 2017; Winker, 2010). Sedentary individuals often initiate breeding before the departure of the migrants, whereas migrants may remain non‐reproductive during the period of overlap (though see Quay, 1989). Thus, reproductive residents would encounter potential mates that exhibit varying levels of readiness to reproduce. Reproductive allochrony may then promote mating preferences based on differences in timing, other morphological divergence, or both. Under these conditions, assortative mating could maintain and even promote divergence between migratory and sedentary populations (Bolnick & Kirkpatrick, 2012; Kirkpatrick & Ravigné, 2002; Winker, 2010). Alternatively, sexual selection and mating preferences may be unnecessary for divergence to occur between migratory populations in seasonal sympatry, as ecological speciation and allochrony may be sufficient to maintain differentiation (Winker, 2010).

We are interested in how mate preferences might act as a mechanism to maintain or promote divergence in seasonal sympatry. In this study, we test whether populations diverging in seasonal sympatry exhibit mating preferences that would encourage assortative mating between diverging populations. We hypothesize that mate choice is adaptive in addition to acting as an important secondary mechanism in maintaining divergence between populations that differ in migratory behavior found in seasonal sympatry. Specifically, we address whether males exhibit mating preferences in seasonally sympatric populations. Researchers have begun to identify the conditions necessary for male mate choice to evolve (Amundsen & Forsgren, 2001; Bel‐Venner, Dray, Allaine, Menu, & Venner, 2008; Bonduriansky, 2001; Pack et al., 2009; Preston, Stevenson, Pemberton, Coltman, & Wilson, 2005; Pryke & Griffith, 2007; Sæther, Fiske, & Kålås, 2001; Tigreros, Mowery, & Lewis, 2014), which raises the possibility that male preferences might also play an important role in maintaining population divergence (Edward & Chapman, 2011).

We tested this hypothesis on the dark‐eyed junco *(Junco hyemalis*), a north‐temperate songbird that diversified approximately 15,000 years ago into multiple, distinct subspecies found across North America (Friis, Aleixandre, Rodríguez‐Estrella, Navarro‐Sigüenza, & Milá, 2016; Milà, McCormack, Castaneda, Wayne, & Smith, 2007). We focused on a migratory subspecies (*J. h. hyemalis;* hereafter “migrants”) that breeds in Canada and winters at our study site in the eastern United States from October until May, and a sedentary subspecies (*J. h. carolinensis;* hereafter “residents”) present year‐round at our study site and throughout the Appalachian Mountains. Both subspecies are classified as “slate‐colored” juncos due to overall similarity in their appearance, but these two subspecies differ subtly in body size, plumage coloration, wing morphology, bill coloration, and migratory phenotype and timing of breeding (Mulvihill & Chandler, 1991; Nolan et al.,). Bill coloration is the most distinct phenotypic difference between migrants and residents. Residents begin to form pairs in early spring before the migrants depart to breed (Nolan et al.,). Migrants tend to lag behind residents in the timing of reproductive development (Fudickar et al., 2016; Kimmitt & Ketterson, 2018). Divergence in migratory behavior and resulting differences in reproductive timing can promote assortative mating and mating preferences between seasonally sympatric populations (Winker, 2010).

To address the question of whether male juncos exhibit mate preferences in seasonally sympatric populations, we conducted simulated courtship interactions (SCIs) in which we presented a free‐living resident male with a live, caged resident or migrant female on his territory and quantified his courtship behaviors (Reichard, Kimmitt, Welklin, & Ketterson, 2017). We predicted that if resident males have a preference for resident females in seasonal sympatry, then males presented with a resident female would spend more time courting and display more vigorously compared to those presented with a migrant female.

## 2 METHODS

### 2.1 Study site and territory mapping

Resident and migrant dark‐eyed junco populations co‐exist in the Appalachian Mountains in winter and are observed in mixed flocks during this time (Cristol et al., 2003; Nolan et al., ). Dark‐eyed juncos are socially monogamous: males defend territories and form pair bonds with one female during each breeding season (Nolan et al., ). Residents begin defending territories and forming pair bonds in mid‐ to late‐March prior to the departure of the migrant subpopulation. Migrants remain in flocks until their departure in mid‐April for the breeding grounds. Resident females caught between 25 March and 11 April had significantly larger ovaries than migrant females (A.A. Kimmitt, unpublished data). In this subset of individuals, no females were ready to lay eggs, however, the earliest egg 1 date in the resident population is April 8 (E.D. Ketterson, unpublished data), suggesting that in some years, residents will initiate egg laying prior to the departure of migrant females.

We conducted our study at Mountain Lake Biological Station (MLBS) in Pembroke, VA (37°22′N, 80°32′W) and the surrounding Jefferson National Forest (Chandler, Ketterson, Nolan, & Ziegenfus, 1994). Migrants typically arrive at MLBS starting in mid‐October, and co‐exist with the residents until their spring migration in mid‐April. There is no known interbreeding between migrants and residents at this study site. The study was conducted early in the resident breeding season in 2016.

At the beginning of the breeding season, all residents on the study site were caught using mist nets and Potter traps and banded with distinctive color combinations. Using a recording of a junco long‐range song, we mapped territory boundaries by starting at the presumed center of the focal male's territory and moving the playback toward the territory edges until the male ceased to follow the playback or had an aggressive encounter with a neighboring male. Territories were mapped at the beginning of the season and assessed periodically during the experiment to adjust for any instability in boundaries.

### 2.2 Simulated courtship interactions (SCIS)

We used five unique resident and five unique migrant females as live female stimuli in the SCIs. Females were held in separate compartments in an outdoor aviary at MLBS. Females had been caught in the Jefferson National Forest in years prior to this experiment and had been used for previous experiments; females were in captivity for 18–28 months prior to the initiation of the trials.

Between 19 April and 13 May in 2016 (i.e., early breeding season), we conducted SCIs on thirty, free‐living resident male juncos. To assess whether males preferred females based on her migratory strategy (i.e., resident or migrant), we presented males with either a resident female (*n* = 15) or migrant female (*n* = 15) paired with a playback of a subspecies‐specific precopulatory trill, a signal of female sexual receptivity (see below). All SCIs were ten minutes long and were conducted between 06:00 and 12:00 EST.

Female stimuli were selected in a random‐stratified order and caught each morning in the aviary before the trials. The female was placed in a cube cage (18 × 18 × 18 cm) in the center of the focal male's territory and covered with camouflage fabric until initiation of the trial. We placed a Pignose speaker (Model No. 7‐100) attached to an Apple iPhone 5 next to the female. A shotgun microphone (Audio‐Technica AT835b) was mounted on a tripod approximately 1‐meter from the female and connected to a Marantz digital recorder (model PMD660) to record any songs produced by the male during the trials.

To attract the focal male to the female, we used a playback consisting of a female trill broadcast every 10 s. We standardized the playback to 90 dB at a distance of 1 m from the speaker using a Radio Shack Digital Sound Level Meter (Model No. 33‐2055). Once the targeted male moved within 10 meters of the female stimulus, we initiated a separate trial playback, which consisted of a female trill every 30 s, played at 70 dB to mimic the natural amplitude of female vocalizations for ten minutes (Reichard, Rice, Schultz, & Schrock, 2013). All males were sighted for unique color combinations to confirm that the trial male was the target individual or a unique individual.

Observers sat 10–15 m away from the lure female. Using a lapel microphone, the first observer, dictated the focal male's courtship behaviors, including song (long‐range song [count] and short‐range song [time spent singing]), time spent with feathers erected (ptiloerection; PT; presence/absence), and time spent tail spreading (TS; presence/absence); the seconded observer dictated male's activity including time spent within 5 meters of the lure cage, and the male's closest approach to the female stimuli. The first observer was blind to the female stimulus migratory strategy at the initiation of each trial; the blindness was effective in the trial because the first observer was new to the study system and it is difficult to differentiate bill coloration of females from a distance. Both observers then scored the audio trial recordings and discussed until they concluded the same start and end times for behaviors.

### 2.3 Design of the playback tapes

We used recordings of precopulatory trills from three resident and three migrant females to create playbacks for our SCIs. To limit pseudo‐replication of the playback stimulus, we generated additional playback tapes using the “Stretch” function in Adobe Audition CS6 (Adobe Systems, San Jose, CA, USA) to alter the trill duration and frequency bandwidth of each trill by ±2.5% and ±5% of the original values (Reichard et al., 2017). All trills were used for one SCI, with the exception of one trill that was used for two SCIs.

### 2.4 Statistical analyses

We used generalized linear mixed models (GLMMs) to test for a relationship between each of the male courtship behaviors and the subspecies of the live female stimulus (chi‐square test statistics). All statistical analyses were conducted in R using glmer function in the lme4 package (version 3.2.0). There was no model that fit the distribution of the closest approach variable, so it was excluded from analysis. Based on the distribution of the data of the remaining five behaviors, we used a Gaussian model including link = log for three behaviors (short‐range song, tail spread, and long‐range song) and link = sqrt for the other two behaviors (ptiloerection and time spent within 5 meters of the female stimulus). Each model was designed as a random intercepts model, as we included female migratory strategy as a fixed effect and the female stimulus ID as a random factor. We then used the ANOVA function (ANOVA () in R) for each model to produce a chi‐square value and p‐value. We used the estimated marginal means for migrants and resident female stimuli in each model in order to calculate the effect size (Cohen's *d*). We used a correction factor in the Cohen's *d* calculations because the sample size was <50 (Durlak, 2009). We followed the general guidelines for interpreting Cohen's *d*, in which a small effect = 0.2, a medium effect = 0.5, and a large effect = 0.8 (Durlak, 2009).

## 3 RESULTS

**Figure 1:**
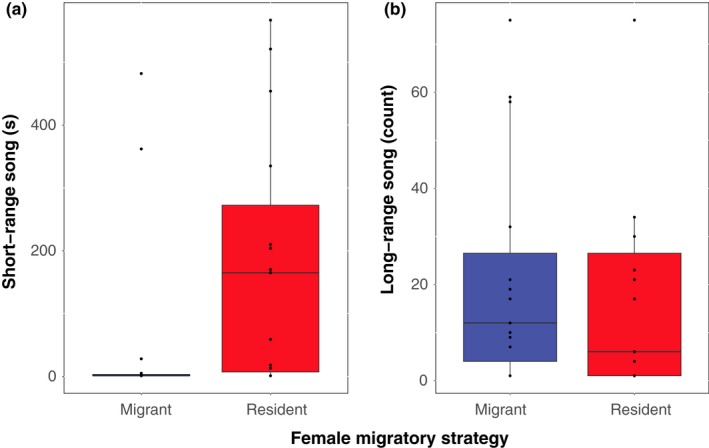
Songs during SCIs (a) duration of short‐range song; GLMM, Lure ID = random effect; *N*_M_ = 15, *N*_R_ = 15, *p* = 0.00123 (b) Count of long‐range song; GLMM, Lure ID = random effect; *N*_M_ = 15, *N*_R_ = 15; *p* = 0.885

**Figure 2:**
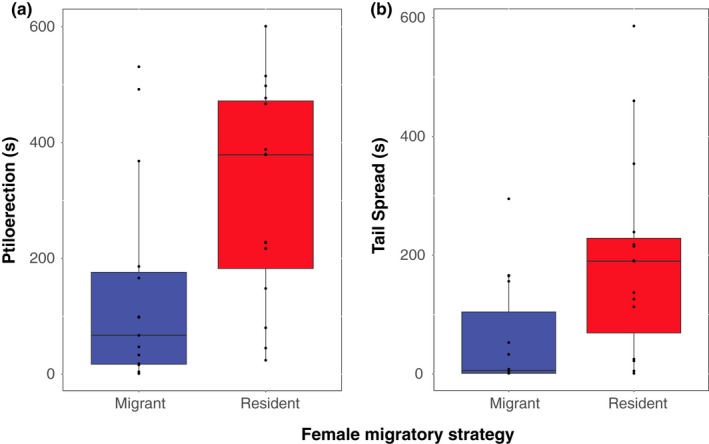
Visual Displays during SCIs; (a) Duration of ptiloerection; GLMM, Lure ID = random effect; *N*_M_ = 15, *N*_R_ = 15, *p* = 0.01 (b) Duration of tail spread; GLMM, Lure ID = random effect; *N*_M_ = 15, *N*_R_ = 15; *p* = 0.012

**Figure 3:**
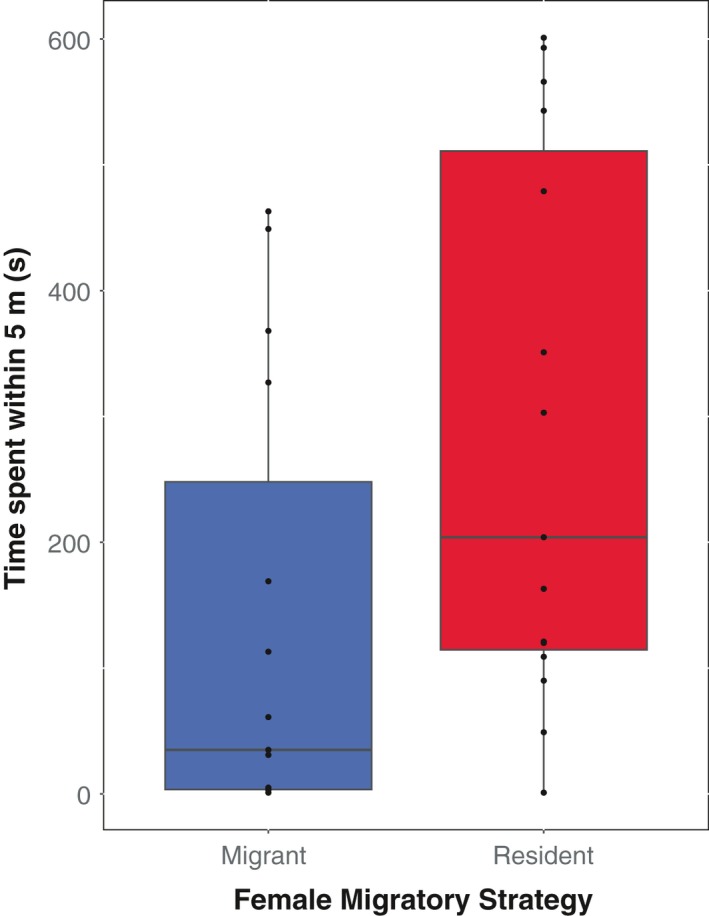
Duration of time spent within 5 meters of the female stimulus; GLMM, Lure ID = random effect; *N*_M_ = 15, *N*_R_ = 15, *p* = 0.0117

Resident males presented with resident females courted more heavily than males presented with migrant females in four of five quantified courtship behaviors (Table 1). Males sang significantly more short‐range song (Figure 1a). Males also exhibited more visual displays, spending significantly more time with their body feathers erected (ptiloerection) (Figure 2a) and significantly more time with their tails spread (Figure 2b). Males also spent more time within five meters of the female (Figure 3). There was a high effect size of female migratory strategy for all significant behaviors, with the exception of ptiloerection, in which there was a medium effect size (Table 1). For the exception, males did not differ in time spent singing long‐range song when presented with a resident or migrant female (Figure 1b).

**Table 1:** Summary of random intercepts generalized linear mixed model outputs, estimated marginal means, and effect size

Behavior	Estimated marginal means (EMM) ± *SE*		Chi‐square	*df*	*p*‐Value	Cohen's *d*
	Migrant	Resident				
Short‐range song (sec)	1.80 ± 0.70	4.97 ± 0.68	10.44	1	**0.001**	1.11
Long‐range song (count)	2.67 ± 0.38	2.75 ± 0.37	0.02	1	0.885	0.05
Ptiloerection (sec)	11.28 ± 1.53	16.84 ± 1.53	6.62	1	**0.01**	0.88
Tail spread (sec)	3.14 ± 0.56	5.13 ± 0.56	6.36	1	**0.012**	0.87
Time within 5 m (sec)	10.87 ± 1.82	15.93 ± 1.82	3.88	1	**0.049**	0.68

Notes

Bold text indicates significant p‐values.

## 4 DISCUSSION

The evidence presented here supports the hypothesis that males exhibit a mating preference when populations differ in migratory behavior. We found that resident males exhibited a courtship preference for females from the same resident population over females from a migratory population in early spring when resident and migrant females overlap in distribution. Resident males spent more time singing a courtship song (short‐range song) and displaying (i.e., ptiloerection and tail spreading) when presented with a resident as compared to a migratory female. Males also spent more time within 5 m of a resident female than a migrant female.

Previous research in other species has shown that male mate choice can be measured by testing for a change in courtship intensity directed toward a given female (Edward & Chapman, 2011). We were unable to measure a change in courtship intensity because of known order effects in behavioral trials (i.e., males always courted the first female presented to him more intensely, regardless of whether the female was a migrant or resident [A.A. Kimmitt unpublished data]. These results of differential courtship effort directed toward either a resident or migrant female across a sample of males suggest that it might be adaptive for males to exhibit mate preferences in seasonal sympatry. Male mate choice in turn thus seems a likely component of assortative mating between diverging populations.

### 4.1 Seasonal sympatry may facilitate evolution of male mating preference

In seasonal sympatry, male mate choice may contribute to assortative mating between populations, as males will encounter potential mates that vary in breeding phenology and morphology. Sedentary populations are typically found at lower latitudes than the breeding range of the migrants (Winker, 2010), enabling populations to overlap during the wintering season. Sedentary individuals often become reproductive as soon as local weather cues and environmental conditions are appropriate for breeding and rearing offspring (Robinson et al., 2009), whereas migrants are likely to be more reliant on predictable cues, such as photoperiod, for timing of migration and reproduction (Ramenofsky et al., 2012).

Migrants often overlap with sedentary populations in early spring after sedentary individuals have initiated their breeding season (Winker, 2010). For example, migratory Red‐eyed Vireos overlap with a sister species, Yellow‐green Vireos, during the first month of the latter's breeding season. Similarly, after Bicknell's Thrushes have initiated egg‐laying, a closely‐related migratory species, Gray‐cheeked Thrushes, remain in sympatry in their breeding range (Winker, 2010). Seasonal sympatry presents an opportunity for interbreeding between migratory and sedentary populations, but interbreeding is uncommon despite suitable conditions for reproduction (Winker, 2010).

Divergence in seasonal sympatry is likely based on population differences in reproductive timing, which can facilitate assortative mating. The timing of reproduction in differentiating migratory populations is often based on utilizing different resources (e.g., resources on the wintering grounds or resources at the migratory breeding ground destination) leading to a key component of seasonal sympatry: reproductive allochrony (Winker, 2010). When seasonally sympatric populations are reproductively allochronic, male mate preferences for females with similar breeding phenology and morphology might additionally maintain or reinforce population divergence and, ultimately, speciation. Our data are consistent with our prediction that males should exhibit mate preferences when populations exhibit seasonal sympatry (Edward & Chapman, 2011).

Males are likely to experience a direct fitness cost to courting and mating with migrant females. Some evidence suggests that females of some migratory species may be inseminated by males and then store sperm during spring migration (Quay, 1989). Passerines, however, tend to exhibit short durations of sperm storage, and sperm from inseminations prior to or during migration are unlikely to be viable when females reach the breeding grounds (Briskie, 1996). Assuming local adaptation, even if courtship and mating are successful, offspring produced are likely to exhibit an intermediate, suboptimal phenotype, such as mistiming of reproduction or migration (Helbig, 1991; Price, 2008; Winker, 2010). Overall, resident males that exhibit mate preferences during seasonal sympatry should be selected for due to higher reproductive fitness as they are more likely to utilize time courting reproductive females. In populations that differ in migratory behavior, divergence may be enforced or maintained by male mating preferences.

Studying male mate preference in the context of seasonal sympatry, however, is only half of the equation as female mate preference might also shape divergence in seasonal sympatry. Future studies should examine whether female mating preference is present in seasonal sympatry, how female mate preferences interact with male mate preferences, and the potential role of female mate preference in maintaining divergence between populations that differ in migratory behavior.

### 4.2 Other factors that may contribute to mating preferences

An additional prediction for why male mate choice is present in seasonally sympatric populations is that phenotypic divergence resulting from disruptive selection alone may favor the evolution of sexually selected traits. Sexually selected traits may encourage assortative mating based on population recognition cues (van Doorn, Edelaar, & Weissing, 2009; Winker, 2010) and maintain population divergence via mate choice as a prezygotic isolating barrier (Turbek et al., 2017). Divergence in sexually selected traits that are important for mate choice are common among birds (Price, 2008; Winker, 2010). Assortative mating and mate choice might drive or maintain population divergence even in cases where there is no current geographic or temporal isolation (Turbek et al., 2017).

Divergence between populations that differ in migratory behavior, however, is more likely driven by ecological factors and resulting allochronic differences, whereas sexual selection and mate choice likely have additive divergent effects (Winker, 2010). Mate choice then could be based on reproductive timing alone. Individuals may utilize behaviors, such as song or precopulatory displays that reflect an individual's reproductive condition to choose mates with a similar migratory strategy. Additionally, the underlying genetics for mate preferences (Hendry & Day, 2005; Winker, 2010) may be simply linked to the loci under selection for phenology (Winker, 2010).

It is less clear how males are able to distinguish between resident and migrant females. Differences in breeding phenology might lead to differences in female behavior (e.g., flocking behavior, receptivity to courtship), but males may also differentiate between females based on morphological differences, such as plumage and bill coloration (Mulvihill & Chandler, 1991; Nolan et al.,). Morphological traits that have diverged along with or after population divergence in migratory behavior could indicate the female's population. If populations have diverged in sexually selected or mate recognition traits, they may also have diverged in their preferences for those traits (Price, 2008). Overall, morphological differences are limited in these populations, which is typical in species diverging in phenology or migratory behavior, because allochronic differences may be sufficient to induce speciation alone (Winker, 2010). Males could also detect behavioral differences (Searcy, 1992) or olfactory differences (Whittaker et al., 2011, 2010 ) that indicate that a female is in reproductive condition.

One limitation of our study is that we cannot detect if males are utilizing female morphological traits and/or female vocalizations to make mate choices. Since we are unable to isolate reproductive timing from morphological traits in the populations, we cannot determine if reproductive timing or morphological traits played a more significant role in male mate preferences. We also did not record the behavior of female stimuli during the trials, so we are unable to determine if migrant and resident females responded differently to male courtship effort. Live female stimuli, however, rarely vocalize or solicit copulation from the focal male (Reichard et al., 2017). Males are likely responding in some way to morphology as indicators of either characteristics of a female of his subspecies or of the female's reproductive condition (i.e., only females that look like residents should be reproductive). We included the female's individual identification as a random factor in our models to ensure that individual differences in response to male courtship did not drive the differences in male's courtship effort observed in this study. While we conclude that male mating preferences based on differences in breeding phenology are present in seasonal sympatry, and therefore likely maintaining divergence, more research is needed to isolate the relative importance of differences in timing and morphology.

### 4.3 Using migratory divides to better understand assortative mating in seasonal sympatry

Although research is limited in addressing assortative mating in seasonal sympatry, more extensive research has focused on assortative mating in similar patterns of allochrony in migratory divides. In migratory divides, different populations of one species may migrate in the fall from common breeding grounds to different wintering grounds of varying distance from the breeding ground; this can lead to allochrony based on differences in arrival time on the breeding grounds (Irwin & Irwin, 2005). These differences in arrival timing, and consequent differences in reproductive timing, could enforce a reproductive barrier to interbreeding between early and late breeders (Coyne & Orr, 2004; Irwin & Irwin, 2005; Rolshausen, Segelbacher, Hobson, & Schaefer, 2009). For example, based on isotopic data, individual European Blackcaps (*Sylvia atricapilla*) form pair bonds with individuals that migrate a similar distance from the breeding grounds (Bearhop et al., 2005). Individuals choose to mate assortatively based on temporal barriers. Assortative mating between populations also selects against hybrid phenotypes, as hybrids typically inherit intermediate migration directions and distances that may also negatively affect timing of migration and breeding (Bearhop et al., 2005).

Assortative mating based on differences in breeding phenology, and resulting in limited gene flow, might also maintain or reinforce divergence in morphology between populations (Rolshausen et al., 2009). Populations that differ in migratory distance and direction likely also differ in wing and bill shape, as well as bill and plumage coloration (Rolshausen et al., 2009). Divergence in morphology between the two populations might reinforce population recognition and further divergence between the migratory groups (Rolshausen et al., 2009). Similarly, in seasonal sympatry, allochrony might accelerate divergence of sexually selected and mate recognition traits between migrant and resident populations.

### 4.4 Implications for the effects of climate change

Our results have interesting implications for populations that may experience climate‐mediated secondary contact as a result of range shifts and changes in breeding phenology due to climate change (Chunco, 2014; Root et al., 2003; Visser & Both, 2005). If assortative mating in seasonal sympatry is based on divergence in sexually selected traits, then speciation may still be favored despite climate‐mediated shifts in phenology. Alternatively, if assortative mating is based on allochrony alone, then it is less likely that divergence will persist. To better understand how climate change will affect speciation, future research should focus on identifying reproductive timing or sexually selected traits as the primary drivers of mate preference in seasonally sympatric or allochronic populations.

## ETHICAL APPROVAL

All institutional and national guidelines for animal use and care were followed. This study was conducted with the approval of the Indiana University Institutional Animal Care and Use Committee (#15‐026‐02).

## CONFLICT OF INTEREST

We have no competing interests.

## AUTHOR'S CONTRIBUTIONS

A.A.K. conceived and designed the study, collected field data, participated in data analysis and statistical analysis, and was the primary author of the manuscript. S.L.D. collected field data, participated in data analysis, and participated in editing the manuscript. D.G.R. helped to conceive and design the study and participated in editing the manuscript. E.D.K. helped conceive and design the study, participated in editing the manuscript, and provided funding and resources. All authors approved this submission.

## DATA ACCESSIBILITY

Supporting behavioral data and R code for models available from the Dryad Digital Repository: https://doi.org/10.5061/dryad.2fb68d1.
